# Epithelioid glioblastoma exhibits a heterogeneous molecular feature: A targeted next-generation sequencing study

**DOI:** 10.3389/fonc.2022.980059

**Published:** 2022-11-24

**Authors:** Rui Pan, Xiaotong Wang, Ru Fang, Qiuyuan Xia, Nan Wu, Qiu Rao

**Affiliations:** Department of Pathology, Affiliated Jinling Hospital, Medical School of Nanjing University, Nanjing, China

**Keywords:** glioblastoma, epithelioid glioblastoma, BRAF V600E, molecular genetics, central nervous system tumour

## Abstract

**Introduction:**

Epithelioid glioblastoma (eGBM) is one of the rare glioblastoma (GBM) variants in the current World Health Organization (WHO) categorization of central nervous system (CNS) tumours. However, the diagnostic basis and molecular features of eGBM have not been clearly defined to date. In this study, we aimed to molecularly characterize these tumours.

**Methods:**

The clinicopathological, molecular, and immunohistochemical characteristics of 12 cases of eGBM were investigated.

**Results:**

The tumours were found to be made up of epithelioid and rhabdoid cells when examined under a microscope. Six cases (50%) harboured the BRAF V600E mutation, and NF1 mutation was detected in 2 eGBM cases (16.7%). CDKN2A/B homozygous deletion was seen in 5 cases (41.7%). TP53 mutation was recognized in 2 instances (16.7%), and TERT promoter mutation was recognized in 5 cases (41.7%).

**Discussion:**

eGBM is characterized by high molecular heterogeneity and has molecular overlaps between low-grade gliomas. Moreover, rather than being a variant or entity, the biological significance of the "epithelioid" appearance may be reduced to a simply morphological pattern. In order to target the proper treatment to suitable patients, molecular stratification via genome-wide molecular profiling will be crucial.

## Introduction

GBM is the extremely frequent and aggressive tumour of the human brain. Epithelioid glioblastoma (eGBM) is the rare type of GBM variables in the 2021 WHO CNS tumours classification. This entity is mostly made up of epithelioid cells with abundant cytoplasm, eccentrically placed nuclei, and prominent nucleoli (1). Due to the lack of particular immunohistochemical or molecular markers for eGBM, diagnosis can be difficult. The *BRAF V600E* mutation has been identified in eGBMs at a relatively great frequency, despite being rare in conventional GBM (54%) (2–5). Moreover, low-grade glioma components in eGBM were reported in recent studies, and a few eGBM patients were previously diagnosed with pleomorphic xanthoastrocytoma (PXA) (6–9). Therefore, several studies have suggested that eGBM and PXA may be either the same entity or closely related (6, 10–13).

eGBM is commonly considered more devastating than classical GBM and has a higher molecular heterogeneity (12, 14). Nevertheless, the clinical features, pathological results and molecular characteristics of eGBM are still poorly understood. Moreover, the diagnostic basis and molecular features of eGBM have not been clearly defined to date. Wide panels of molecular and immunohistochemical markers are required to achieve the correct diagnosis. We described the clinicopathological and molecular characteristics of 12 eGBMs and discussed their molecular genetic features.

## Methods

### Data collection

The Institute Research Ethics Committee of Jinling Hospital approved this study. Slides from glioblastomas were retrieved from 2014 to 2022 surgical pathology files of the authors’ institution (Affiliated Jinling Hospital, Medical School of Nanjing University) and were involved in the study if they were diagnosed as eGBM on the basis of characteristic morphological and molecular features. Two pathologists performed a blinded review of the pathological materials according to the pathological and molecular definition of eGBM in the 2021 WHO categorization of CNS tumours. Thirteen GBM cases were consistent with epithelioid morphology. Case 13 was eliminated from the series because of the involvement of an *IDH1* mutation. In total, 12 eGBMs were gathered in this study. The clinical, radiological and pathological data were obtained from the Department of Pathology, Affiliated Jingling Hospital, Medical School of Nanjing University. Reviewing electronic health records and attempting to contact referring pathologists and clinicians yielded clinical and demographic follow-up information.

### Immunohistochemistry

Tumour tissues were embedded in paraffin after being fixed in 10% formalin. Sections were cut out at 3 μm thickness and immunohistochemically stained with conventional antibodies as well as several available commercially antibodies against gene expression targets identified throughout the gene expression analysis. The following proteins were chosen as targets: GFAP (MAB-0764, 1:150, Maixin Bio (MXB)), INI1 (ZA-0696, ready-to-use, Zhongshan (ZSGB)), IDH1 (ZM-0447, ready-to-use, ZSGB), BRAF V600E (790-5095, ready-to-use, Roche), CKpan (kit-0004, 1:200, MXB), ATRX (MAB-0855, ready-to-use, MXB), EMA(ZM-0095, ready-to-use, ZSGB) and TP53 (ZM-0408, 1:200, ZSGB).

TP53 immunostaining was identified as a missense mutation when higher than 10% nuclear positivity was exist (15). Immunostaining was defined as a frameshift when tumour cells demonstrated a full absence of nuclear staining, and intrinsic control cells showed focal nuclear staining (16, 17). Both missense and frameshift mutations were considered TP53 mutants (15, 16). Internal negative or positive controls, including endothelial cells and/or trapped cortical neurons, were identified in all immunostainings.

### Targeted next-generation sequencing

Sequencing of a 425-gene panel was performed on the cases (**Supplementary Table S1**). Nucleic acid isolation for NGS was performed on formalin-fixed paraffin-embedded (FFPE) tumour tissue from a microdissected representative block. Following the generator’s instructions, five 10 μm tumour slices were utilized for DNA extraction utilizing the QIAamp DNA FFPE Kit (QIAGEN, Valencia, CA, USA). The quality of the DNA was determined using spectrophotometry with absorbance at 230, 260, and 280 nm, and the DNA was measured using Qubit 2.0. Sequencing libraries were created utilizing the KAPA Hyper Prep Kit (KAPA Biosystems) based on the manufacturer’s recommendations for various specimen types.

In summary, end repair, A-tailing, and ligation with indexed adapters were applied to 1 g of fragmented genomic DNA prior to size selection with Agencourt AMPure XP beads (Beckman Coulter). For hybridization-based target enrichment, the GeneseeqOneTM pan cancer gene panel (425 cancer-relevant genes, Geneseeq Technology Inc.) and the xGen Lockdown Hybridization and Wash Reagents Kit were utilized (Integrated DNA Technologies). Libraries captured by Dynabeads M-270 (Life Technologies) were amplified in KAPA HiFi HotStart ReadyMix (KAPA Biosystems), and their quantities were assessed by qPCR through KAPA Library Quantification Kit (KAPA Biosystems). On the Illumina HiSeq4000 platform, target-enriched libraries were sequenced with 2×150 bp paired-end reads. The Burrows-Wheeler Aligner was applied to match the sequencing dataset to the reference hg19 genome (Human Genome version 19). Sequencing data collected were demultiplexed by bcl2fastq (v2.19), analysed by Trimmomatic (18) to eliminate low-quality (quality <15) or N bases, and afterwards aligned to the reference hg19 genome (19). By using Picard (found at https://broadinstitute.github.io/picard/), PCR duplicates were eliminated. For base quality assurance and local realignments around indels, the Genome Analysis Toolkit (GATK) was used (20).SNPs and indels were identified by VarScan2 (21) and Haplotype Caller/Unified Genotyper in GATK, with a mutant allele frequency (MAF) cut-off of 0.5% for tissue cases and a least of three optimal mutant reads. Frequent variants were eliminated utilizing dbSNP and the 1000 Genome Project. An internal list of repeated sequencing errors generated from more than 10000 normal control cases sequenced on the same platform was used to further filter the resulting somatic variants. FACTERA identified gene fusions (22), and copy number variations (CNVs) were measured with ADTEx (23). For tissue samples, the log2 ratio cut-off for copy number gain was given as 2.0. All specimen types were used to detect copy number loss using a log2 ratio cut-off of 0.67. The thresholds were established from the absolute CNVs found by droplet digital PCR, which was used for earlier assay validation (ddPCR). FACETS (24) was used to estimate allele-specific CNVs with a 0.2 drift cut-off for unstable joint segments. By splitting the size of drifted segments by the overall segment size, the chromosomal instability’s percentage (CIN) was recorded.

## Results

### Clinical data

**Figure 1:**
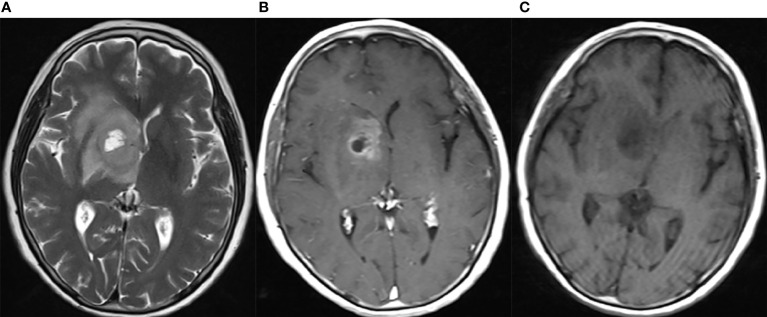
Neuroradiological findings for eGBM case 11. Neuroradiological results for eGBM case 11. **(A)** A heterogeneous lesion with necrosis and perilesional oedema on T1 in the right basal ganglia with significant midline shift, 23 mm × 28 mm × 17 mm in size. **(B)** A heterogeneous lesion with perilesional oedema (T2). **(C)** A rim-enhancing mass with perilesional oedema (T1-weighted enhanced).

The clinical and histopathological data of eGBMs were tabulated and are presented in **Table 1**. There were 9 female and 3 male cases with ages varying from 28 to 70 years. The frontal lobe involving was 3, the temporal lobe involving was 5, the parietal lobe involving was 2, and the basal ganglia was 2. The most common symptoms were headaches and seizures. Radiological examination demonstrated gadolinium-enhancing, comparatively circumscribed lesions with significant perilesional oedema and central necrosis in all cases (**Figure 1**). In 1 case, there was a midline shift (8.33%). All patients had gross total resection. After surgery, 7 patients (58.3%) received radiation or chemotherapy. One patient received targeted therapy (case 12), and have not demonstrated tumour recurrence or metastatic disease to date. The follow-up period varied from 1 to 30 months. One patient was lost to followed-up. At the time of data cut-off, 4 cases developed local recurrences, and succumbed to complications (case 4, case 5, case 6 and case 7). One case developed a pulmonary metastasis (case 2). No radiological or histological evidence of cerebrospinal fluid dissemination was found.

**Table 1:** Summary of the clinical parameters of 12 eGBM patients.

Case	1	2	3	4	5	6	7	8	9	10	11	12
Age/Sex	F*/58	F/59	F/51	M*/53	F/61	F/69	M/30	F/42	M/55	F/62	F/70	M/28
Location	Right Parietal lobe	Right Temporal lobe	Left Frontal lobe	Left Frontal lobe	Right Temporal lobe	Left Frontal lobe	Left Temporal lobe	Right Temporoparietal	Bilateral Frontal lobe	Right Basal ganglia	Left Basal ganglia	Right Parietal lobe
Symptoms	Myodynamia weakness	Headache	Headache	Slurred speech	Headache	Headache, slurred speech	Headache, seizures	Seizures	Headache, memory loss	Limited limb mobility	Headache, slurred speech	Headache, seizures
Follow up in months	24 (Alive)	30 (Alive)	(Lost to follow-up)	12 (Dead)	15 (Dead)	10 (Dead)	1 (Dead)	8 (Alive)	16 (Alive)	12 (Alive)	18 (Alive)	28 (Alive)
Resection type	GTR*	GTR	GTR	GTR	GTR	GTR	GTR	GTR	GTR	GTR	GTR	GTR
Chemotherapy/radiation therapy	Radiation therapy	Radiation therapy	(Lost to follow-up)	Radiation therapy	Radiation therapy	None	None	None	Chemotherapy	None	Chemotherapy	Radiation therapy, Chemotherapy
Microvascular proliferation	Present	Present	Present	Present	Present	Present	Present	Present	Present	Present	Present	Present
Epithelioid cells	≥30%	≥30%	≥30%	≥30%	≥30%	≥30%	≥30%	≥20%	≥20%	≥20%	≥20%	≥30%
Necrosis	Confluent	Confluent	Confluent	Confluent	Confluent	Confluent	Confluent	Confluent	Confluent	Confluent	Confluent	Confluent
Recurrence	None	None	None	Recurrence	Recurrence	Recurrence	Recurrence	None	None	None	None	None
Metastasis	None	Pulmonary	None	None	None	None	None	None	None	None	None	None
Cerebrospinal fluid dissemination	None	None	None	None	None	None	None	None	None	None	None	None

*GTR, gross total resection; F, female; M, male.

### Histopathological findings

**Figure 2:**
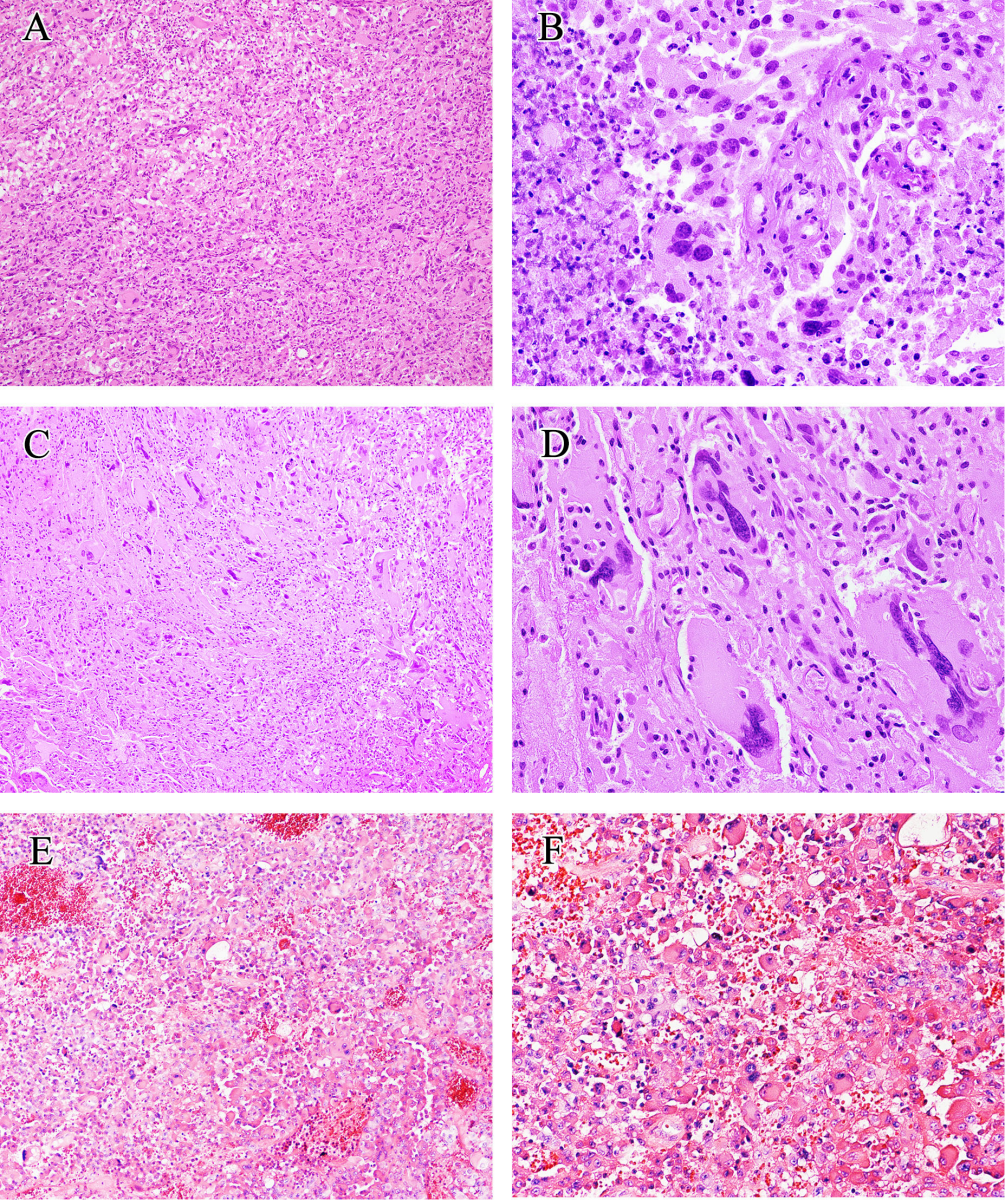
Histological findings of eGBM. **(A)** Patches of epithelioid and rhabdoid cells were presented (×200). **(B)** The tumour showed microvascular proliferation and zonal necrosis, and epithelioid and rhabdoid cells constituted abundant and uniformly eosinophilic cytoplasm and laterally located oval to pleomorphically shaped nuclei. Mitoses could easily be seen (×400). (**C, D**) PXA-like components in eGBM cases showed multinucleated pleomorphic cells, a fascicular arrangement of spindle-shaped cells and single large bizarre cells (×200 and ×400). **(E, F)** Histopathological findings of case 13 (*IDH*-mutant astrocytoma). The tumour presented epithelioid morphology (×200 and ×400).

The histological results are presented in **Table 1** and **Figure 2**. The main notable features of most eGBMs were abundant epithelioid cells and extensive necrosis (**Figure 2**). In all 12 cases analyzed, microscopy revealed eGBM histopathological types (or melanoma or epithelioid-like cells’ sheets with abundant cytoplasm, eccentric nuclei, and loose cohesion). All tumours showed signs of microvascular proliferation, brisk mitotic activity, and necrosis. However, 4 cases had focal areas that resembled PXA (WHO grade 2) appearance (the set of spindled cells forming fascicles, single large bizarre cells, and vacuolated tumour cells with perivascular lymphocytic cuffing).

### Immunohistochemistry

**Figure 3:**
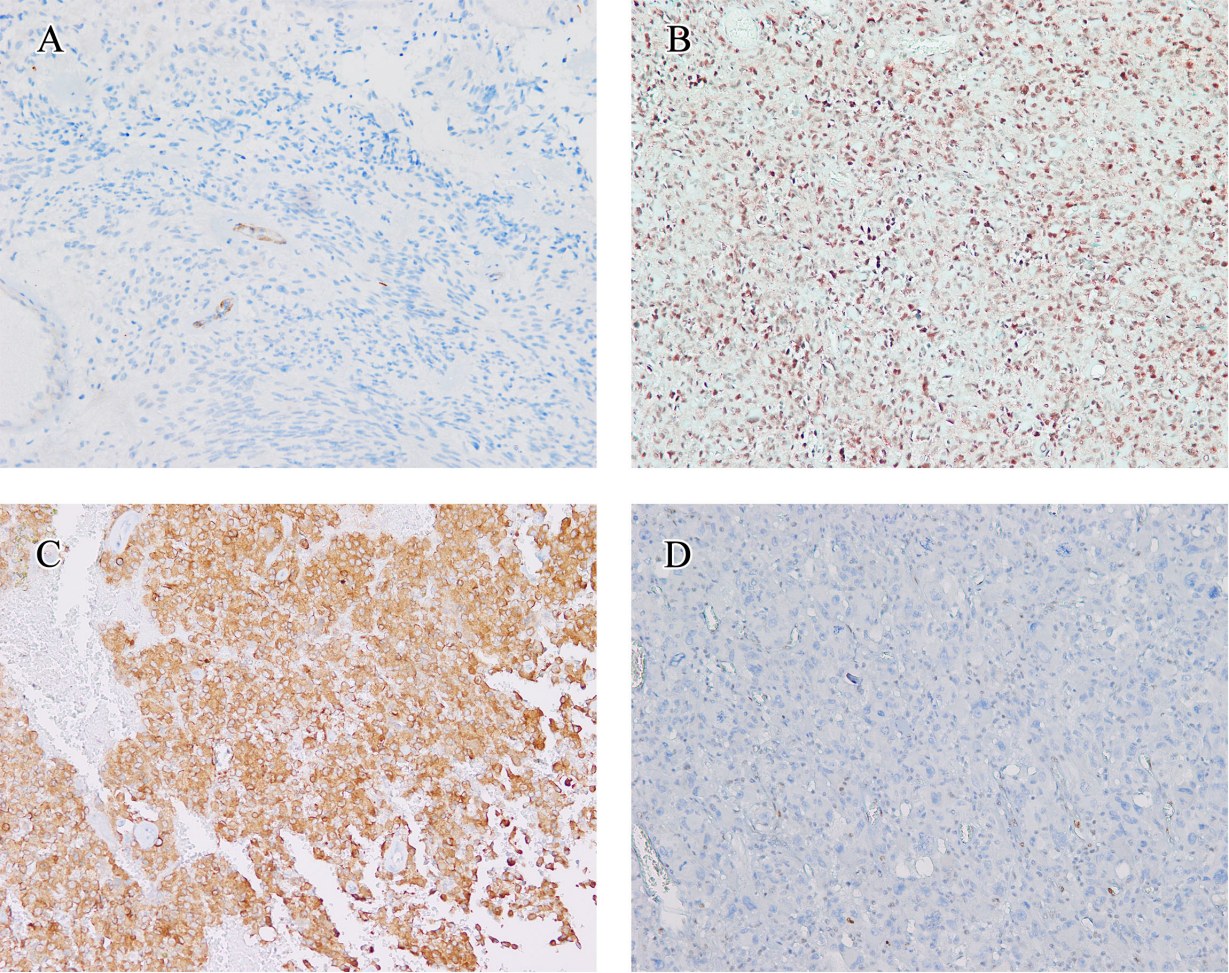
Immunohistochemical findings. The immunohistochemical findings of eGBMs. **(A)** Immunohistochemical studies showed negtive PTEN expression in 2 cases. Vascular endothelial cells provided an internal positive control (×200). **(B)** INI1 staining was universally intact (×200). **(C)** Positive expression of BRAF V600E in eGBM (×200). **(D)** The tumour cells demonstrated a complete absence of TP53 staining and lymphocytes showed TP53 nuclear staining focally (×200).

The immunohistochemistry outcomes are presented in **Table 2** and **Figure 3**. eGBM showed diffuse and strong staining with vimentin. GFAP (glial fibrillary acidic protein) immunoreactivity was diffusely observed in epithelioid cells and lower-grade glioma cells. eGBMs did not show cytokeratin (CK) or epithelial membrane antigen (EMA) staining. The SMARCB1 (INI1) staining was universally intact. Mutant TP53 was observed in 2 cases, and both cases were frameshift mutations. The ATRX loss expression was not observed in any case. IDH1 expression was also not observed in any case. BRAF V600E expression occurred in 50% (6/12) of cases.

**Table 2:** Immunohistochemistry of 12 eGBM cases.

	1	2	3	4	5	6	7	9	10	11	12	13
GFAP*	3+	2+	2+	3+	3+	3+	3+	1+	3+	2+	3+	3+
S-100	3+	3+	–	1+	3+	2+	3+	3+	3+	2+	2+	2+
ATRX	3+	3+	3+	3+	3+	3+	3+	3+	3+	3+	3+	3+
BRAF V600E	–	–	–	–	3+	–	2+	1+	–	–	3+	3+
INI-1*	Intact	Intact	Intact	Intact	Intact	Intact	Intact	Intact	Intact	Intact	Intact	Intact
IDH1	–	–	–	–	–	–	–	–	–	–	–	–
TP53	–	–	Mutated	–	–	–	–	–	–	–	Mutated	–
CK*	–	–	–	–	–	–	–	–	–	–	–	–

*GFAP, glial fibrillary acidic protein; CK, cytokeratini; EMA, epithelial membrane antigen; INI1, SMARCB1.

### Genetic analysis

**Figure 4:**
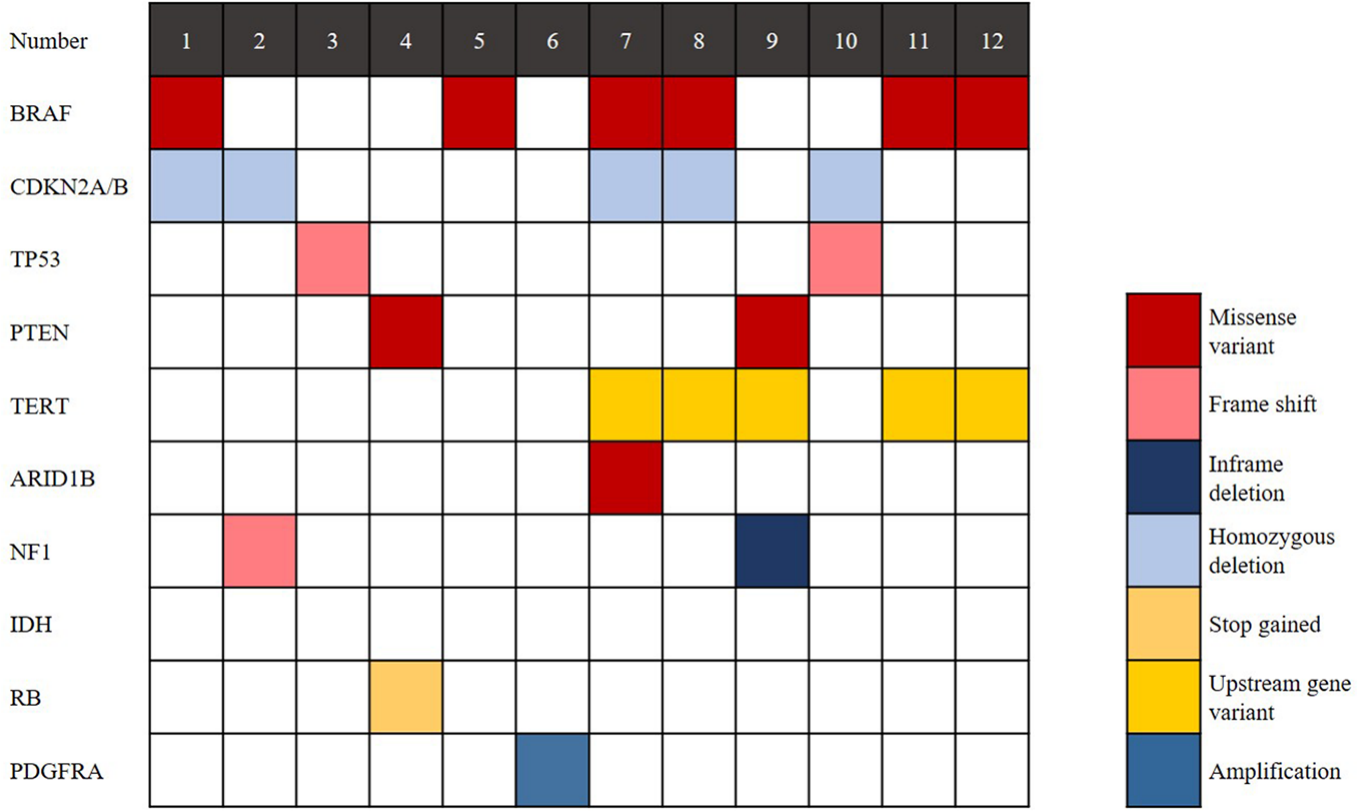
Genomic landscape of eGBM. Clinical and genomic features of 12 eGBM cases. In addition to *BRAF V600E* mutation, eGBM also showed *TP53* mutation, *CDKN2A/B* homozygous deletions and *NF1* mutation.

The findings of genetic analysis are outlined in **Figure 4** and **Supplementary Table S2**. Six cases (50%) harboured the *BRAF V600E* mutation, and *CDKN2A/B* homozygous deletion was seen in 5 cases (41.7%). *TP53* mutation was detected in 2 cases (16.7%), and *TERT* promoter mutation was detected in 5 cases (41.7%). *PTEN* deletion was detected in 2 cases (16.7%). Two of 6 cases without *BRAF V600E* mutation showed *NF1* mutation. *IDH* and *H3 K27M* mutations were not found in any cases. In conclusion, eGBMs are complex and heterogeneous tumours, exhibiting multiple genetic mutations.

## Discussion

Epithelioid glioblastoma is a rare and extremely aggressive variant of GBM. Kepes et al. first characterized it in 1982, and it was suggested as a histological subtype in the WHO classification of CNS tumours in 2021 (25, 26). However, the radiological, histological and molecular signature of eGBM have not been clearly defined (10, 27). In this study, we applied combined NGS, histology, radiology and immunohistochemistry to describe the clinicopathological and molecular characterization of eGBM.

Histologically, eGBMs are dominated by a population of epithelioid cells with focal discohension, eosinophilic cytoplasm, a differentiated cell membrane, and a nucleus placed laterally. The tumour is richly vascularized, involving thick- and thin-walled vessels with microvascular proliferation and hyaline degeneration, and also glomerulus-like vasculature. Extensive palisading necrosis has also been observed in eGBM. Although the exact aetiology and origin of epithelioid cells are unidentified, there have been numerous studies of eGBMs occurring concurrently with PXA, particularly tumours with anaplastic transformation and epithelioid characteristics, or occurring years after initial tumour resection (5, 10). Four eGBM cases in our series also presented PXA-like (WHO grade 2) morphological characteristics focally. PXA-like components (WHO grade 2) coexisting with eGBM demonstrated a spindle-shaped cells with some mono- or multinucleated pleomorphic cells (**Figure 2**). Intercellular reticlin meshwork and perivascular lymphocytic cuffing were noticed. Although eGBM is commonly considered to be a primary/*de novo* lesion, numerous cases of eGBM with a pre- or coexisting lower-grade component have been noted (2, 4, 6, 9, 13, 28). The majority of the lower-grade lesions identified thus far were PXA (WHO grade 2), and a few were low-grade diffuse glioma-like lesions (6–9). We speculate that these unique pathological features may be associated with the molecular heterogeneity.

Consistent with those reported in the literature, half of the eGBMs (50%) in our series were involved in the *BRAF V600E* mutation. *NF1* mutation was detected in 2 eGBM cases (16.7%). The *NF1* mutation was mutually exclusive to the *BRAF V600E* mutation. The codon 600 mutation (V600E) is the main mutation site for the *BRAF* gene, which is located on chromosome 7q34. *BRAF* is the gene that encodes cytoplasmic serine-threonine kinase. Subsequent activation of the mitogen-activated protein kinase (MAPK) signaling pathway occurs through the mutated BRAF protein, which in turn promotes tumourigenesis, cellular proliferation, as well as resistance to apoptosis (3, 14). The *NF1* gene is located on 17q11.2 and encodes a tumour suppressor that works as a GTPase-activating protein to deactivate the RAS/MAPK signalling pathway, finally causing the occurrence of tumours (29, 30). Hence, both *NF1* mutations and *BRAF V600E* mutations contribute to the constitutive stimulation of downstream RAS/MAPK signalling pathways (31–33), which may be associated with unique pathological features similar to eGBM and PXA (30, 34). Several studies have reported that part of *wt-IDH* glioblastomas with *NF1* mutation also presented a xanthomatous histological appearance (34, 35). Consequently, in addition to *BRAF V600E*, *NF1* mutation may be another meaningful biomarker for the diagnosis of eGBMs. However, the proportion of *NF1* mutation in *BRAF V600E* negative eGBMs demands further investigation.

The work of Korshunov et al. has also illustrated the molecular heterogeneity of eGBM (11) (**Table 3**). They identified three distinct, previously described subtypes of tumours by combining data from methylation types, copy number alterations, as well as mutations analysis with outcomes from clinical trials. According to the authors, histopathologically defined eGBM is divided into at least 3 molecularly and biologically distinguishable classifications. Consequently, the outcome that eGBM molecularly shares overlaps with other subtypes of glioblastoma may reduce their epithelioid appearance to a morphological pattern, and decrease the biological significance of it.

**Table 3:** Review of previous studies including mutational analysis.

Author/year	No. of cases	Age/Sex	Necrosis (% of cases)	Follow up in months	MVP* (% of cases)	IDH1	CDKN2A/B	PTEN	Braf V600E	TP53	TERT	NF
Kahanna et al., 2018	7	13~50/M-3 F-4	100%	3~6	28%	None	Not Done	None	28%	Not Done	40%	Not Done
Kleinschmidt et al., 2013	13	10~69/M-9 F-4	92%	5~82	7%	9%	Not Done	Deletion (33%) Monosomy (33%) Negative (33%)	54%	33%	Not Done	Not Done
Alexandrescu et al., 2015	11	2~79/M-9 F-2	93%	2~38	87%	None	Not Done	Deletion (12%) Monosomy (12%)	53%	36% (IHC)	Not Done	36% (IHC)
Korshunov et al., 2020	64	3~67/M-45 F-19	100%	5~72	Not Applicable	None	55%	Not Done	56%	Not Done	38%	Not Done
Ying et al., 2020	15	18~77/M-12 F-3	100%	One week~32	Not Applicable	None	Not Done	Not Done	47%	47% (IHC)	Not Done	Not Done
Debajyoti et al., 2020		3~54/M-12 F-12	96%	5~38	100%	None (IHC)	Not Done	Not Done	52% (IHC)	Not Done	Not Done	Not Done
Our Present study	12	28~70/M-4 F-8	100%	1~30	100%	None	42%	Deletion (17%)	50%	17%	42%	17%

*MVP, microvascular proliferation.

Molecularly, in this series, *TERT* promoter mutation was detected in 41.7% (5/12) of cases. *CDKN2A/B* homozygous deletion was seen in 41.7% of cases and *TP53* mutation was detected in 16.7% of cases. A total of 16.7% of cases were confirmed to have *PTEN* deletion (**Figure 4**). Some reports documented the *TERT* promoter mutation in GBMs, suggesting its role in the aggressive clinical course (4, 36). *TERT* promoter mutation is a poor prognostic indicator in *wt-IDH* gliomas. Moreover, the exitance of *pTERT* mutation partially clarifies the aggressive nature of GBMs, and its correlation with the tumour’s ability to overcome escape apoptosis and replicative senescence (the fundamental steps in tumourigenesis). *CDKN2A* is a tumour suppressor gene located on chromosome 9p21. It encodes the p16 protein, a negative regulator of cell cycle progression. The *CDKN2B* gene is located next to *CDKN2A*. The mutation to either *CDKN2A* or *CDKN2B* will lead to cellular proliferation and the disruption of proapoptotic pathways (37). In *IDH*-mutated gliomas, *CDKN2A* homozygous deletion is a strong adverse prognostic factor (38). *PTEN* is located on 10q23.3 and consists of 9 exons. *PTEN* deletion has been proven to correlate with poor survival in glioblastoma, suggesting that *PTEN* plays a role in patient outcomes (39). In this study, most cases (83.3%, 10/12) showed at least 1 mutation mentioned above, which has been detected frequently in gliomas and associated with poor prognosis. Even though, the prognosis of patients are quite different (**Table 1**), which further illustrates the clinical heterogeneity of eGBM.

Interestingly, case 13 in our study, which exhibited an epithelioid morphology (**Figure 2**), had both the *BRAF V600E* mutation and an *IDH1* mutation. Consistent with the reports of *IDH*-mutated glioblastomas, this patient had a relatively long overall survival of up to 30 months. In consequence, this case should be diagnosed as *IDH*-mutant astrocytoma (WHO grade 4). Accordingly, when high-grade gliomas present epithelioid morphology, the diagnosis of eGBM may not be necessary. Another study also reported that *K3 K27M*-altered gliomas exhibited an epithelioid appearance (10).

In summary, we studied 12 eGBM cases and further described the clinicopathological and molecular features of the tumours. Our study indicates clinical and molecular heterogeneity among eGBMs. We propose that in addition to *BRAF V600E*, *NF1* mutation may be another meaningful biomarker for the diagnosis of eGBMs. Instead of being a variant or entity, the “epithelioid” GBM phenotype might be a histologic subtype. In order to target the proper treatment to suitable patients, molecular stratification *via* genome-wide molecular profiling will be crucial in the upcoming years.

## Data availability statement

The datasets presented in this study can be found in online repositories. The names of the repository/repositories and accession number(s) can be found below: Dryad, doi: 10.5061/dryad.2280gb5w0.

## Ethics statement

Approval for this study was granted by the Institute Research Ethics Committee of Jinling Hospital.

## Author contributions

RP: Methodology, Formal analysis, Data curation, Writing-Original draft preparation. XW: Conceptualization, Formal analysis. RF: Data curation, Visualization. QX: Conceptualization. NW: Conceptualization, Project administration. QR: Conceptualization, Methodology, Project administration. All authors contributed to the article and approved the submitted version.

## Funding

This work was supported by grants from the National Natural Science Foundation of China (81802557 to QX and 81872095 to QR).

## Conflict of interest

The authors declare that the research was conducted in the absence of any commercial or financial relationships that could be construed as a potential conflict of interest.

## Publisher’s note

All claims expressed in this article are solely those of the authors and do not necessarily represent those of their affiliated organizations, or those of the publisher, the editors and the reviewers. Any product that may be evaluated in this article, or claim that may be made by its manufacturer, is not guaranteed or endorsed by the publisher.
